# “Benznidazole-resistance in *Trypanosoma cruzi*: Evidence that distinct mechanisms can act in concert’’ [Mol. Biochem. Parasit. (2014) 193, 17–19]

**DOI:** 10.1016/j.molbiopara.2015.05.006

**Published:** 2015-05

**Authors:** M.C.O. Campos, L.L. Leon, M.C. Taylor, J.M. Kelly

**Affiliations:** a Department of Pathogen Molecular Biology, London School of Hygiene and Tropical Medicine, Keppel Street, London WC1E HT, UK; b Laboratório de Bioquímica de Tripanosomatídeos, Instituto Oswaldo Cruz, IOC, Avenida Brasil 436, Manguinhos, Fundação Oswaldo Cruz, Rio de Janeiro, Brazil

The authors regret that Fig. 1 in this paper contains typographical errors. The benznidazole EC5 values on the *Y*-axis of Fig. 1E and F, and the heading to Fig. 1G, are shown as mM (millimolar). These values should be μM (micromolar).

The authors would like to apologise for any inconvenience caused.

